# Patient Recruitment Into a Multicenter Clinical Cohort Linking Electronic Health Records From 5 Health Systems: Cross-sectional Analysis

**DOI:** 10.2196/24003

**Published:** 2021-05-27

**Authors:** Wendy L Bennett, Carolyn T Bramante, Scott D Rothenberger, Jennifer L Kraschnewski, Sharon J Herring, Michelle R Lent, Jeanne M Clark, Molly B Conroy, Harold Lehmann, Nickie Cappella, Megan Gauvey-Kern, Jody McCullough, Kathleen M McTigue

**Affiliations:** 1 Johns Hopkins University School of Medicine Baltimore, MD United States; 2 University of Minnesota School of Medicine Minneapolis, MN United States; 3 University of Pittsburgh Pittsburgh, PA United States; 4 Penn State College of Medicine Hershey, PA United States; 5 Lewis Katz School of Medicine Philadelphia, PA United States; 6 Geisinger Health Danville, PA United States; 7 University of Utah School of Medicine Salt Lake City, UT United States

**Keywords:** electronic health record, recruitment methods, cohort study design, recruitment, health system, bariatric, surgery, clinical research network, primary care, cohort, enrollment, research, process, efficiency, eligibility

## Abstract

**Background:**

There is growing interest in identifying and recruiting research participants from health systems using electronic health records (EHRs). However, few studies have described the practical aspects of the recruitment process or compared electronic recruitment methods to in-person recruitment, particularly across health systems.

**Objective:**

The objective of this study was to describe the steps and efficiency of the recruitment process and participant characteristics by recruitment strategy.

**Methods:**

EHR-based eligibility criteria included being an adult patient engaged in outpatient primary or bariatric surgery care at one of 5 health systems in the PaTH Clinical Research Network and having ≥2 weight measurements and 1 height measurement recorded in their EHR within the last 5 years. Recruitment strategies varied by site and included one or more of the following methods: (1) in-person recruitment by study staff from clinical sites, (2) US postal mail recruitment letters, (3) secure email, and (4) direct EHR recruitment through secure patient web portals. We used descriptive statistics to evaluate participant characteristics and proportion of patients recruited (ie, efficiency) by modality.

**Results:**

The total number of eligible patients from the 5 health systems was 5,051,187. Of these, 40,048 (0.8%) were invited to enter an EHR-based cohort study and 1085 were enrolled. Recruitment efficiency was highest for in-person recruitment (33.5%), followed by electronic messaging (2.9%), including email (2.9%) and EHR patient portal messages (2.9%). Overall, 779 (65.7%) patients were enrolled through electronic messaging, which also showed greater rates of recruitment of Black patients compared with the other strategies.

**Conclusions:**

We recruited a total of 1085 patients from primary care and bariatric surgery settings using 4 recruitment strategies. The recruitment efficiency was 2.9% for email and EHR patient portals, with the majority of participants recruited electronically. This study can inform the design of future research studies using EHR-based recruitment.

## Introduction

### Background

Recruitment of patients for research using electronic health records (EHRs) has potential to enhance the applicability and efficiency of patient-centered research [1,2]. Despite logistical and ethical challenges with recruiting for research using clinical data sources (eg, EHRs) or clinical communication methods (eg, EHR patient portals) [3], researchers are developing strategies that are responsive to both health system and patient stakeholders [4]. In addition, there is interest in designing research studies that include “real-world evidence” (ie, health care information representative of patients, populations, and health care delivery systems from actual clinical settings [5,6]).

In 2014, the National Patient-Centered Research Network (PCORnet) was launched with funding from the Patient-Centered Outcomes Research Institute (PCORI). PCORnet's infrastructure can support EHR-based recruitment and study implementation [7]. The PaTH Clinical Research Network (CRN) originally brought together 4 academic medical centers in the mid-Atlantic region of the United States to build the infrastructure to share EHR data across health systems so that patient-centered clinical questions could be answered in real-world settings [8]. A fifth academic health center (Geisinger Health System) was added to the network in 2015.

Using EHR data from these 5 health systems, standardized to PCORnet specifications [9], we developed a cohort of primary care and bariatric surgery patients called the “Healthy Lifestyles, Body Weight, and Health Care” cohort. The overall aim of the cohort was to complete online surveys about quality of life, healthy lifestyles, and weight management, which could be linked to selected EHR data. Another explicit goal of the CRN was to utilize several different recruitment methods in order to compare sample demographics and survey response rates by recruitment strategy. In this paper, we describe the enrollment process and then compare 4 different recruitment strategies: (1) in-person recruitment by study staff from clinical sites, (2) US postal mail recruitment letters, (3) secure email, and (4) direct EHR recruitment through secure patient web portals. The objective of this paper was to describe the steps and efficiency of the recruitment process and participant characteristics by strategy.

## Methods

Institutional review board approval was obtained from PaTH's single institutional review board at The Johns Hopkins University School of Medicine, Baltimore, Maryland. We benefitted from the input of patient stakeholders in the design of the recruitment methods and online survey. Participants who enrolled in this study were not offered any compensation.

### EHR-Based Participant Eligibility Criteria

We recruited adult patients from primary care and bariatric surgery clinics to complete surveys and form an EHR cohort from 5 health care systems included in the PaTH CRN—Geisinger Health System, Johns Hopkins Health System, Penn State Milton S. Hershey Medical Center, Temple Health System, and the University of Pittsburgh Medical Center. We identified participants using EHR-based eligibility criteria (ie, the “computable phenotype”). Eligible patients were aged ≥18 years and had a minimum of 2 weight measurements and 1 height measurement recorded between January 1, 2011, and May 31, 2015. Patients in all BMI categories were eligible. Participants were excluded if they were deceased or non–English proficient, as assessed at the time of consent, because the consent form and survey were only available in the English language.

### Participant Recruitment and Setting

Each health system used one or more of the following 4 EHR-based research invitation strategies: (1) in-person recruitment by study staff from partnering outpatient primary care and bariatric surgery clinics; (2) US postal mail recruitment invitation letters using the mailing address listed in the EHR, with an online registration link contained in the invitation; (3) email to potentially eligible participants using the email address stored in the EHR, with an embedded registration link in the email; and (4) secure EHR recruitment messages through the patient web portal with an embedded registration link.

Each health system tailored its own strategy to recruit participants from the large pool of potentially eligible patients identified using the computable phenotype. At each site, local norms for integrating research with clinical practice were followed, leading to differences in the number of patients approached by each strategy per site. Importantly, each site’s study team partnered with specific clinical practices so that recruitment letters were jointly sent from clinical representatives involved in the patients’ care and members of the research team and focused on patients from certain practices. Notably, 2 health systems used direct EHR recruitment through secure patient web portals (sites C and D), while 1 site (site B) was unable to use email because email address was not a data field within the EHR. The content of the recruitment letters and in-person scripts had similar language across all health systems and recruitment strategies.

Each health system had a slightly different recruitment window, but the total recruitment window for all sites was from April 2015 to November 2016. We stopped the recruitment when we met or exceeded the a priori goal of recruiting 1000 participants.

### Consent and Enrollment Process

Regardless of the recruitment strategy or method used to complete the baseline survey, all participants completed a web-based consent form, including an initial “consent quiz” designed to ensure that patients understood that they were agreeing to participate in a research study. Participants consented to complete online surveys and for the research study to access their EHR. Enrollment was determined by consenting and having any data entered into the baseline survey. Participants who were recruited in person used an electronic tablet to access the consent form and complete the baseline survey. For participants recruited through the EHR patient portal, the survey was embedded in the EHR, which required that it had to be completed and “submitted” in order to be accessible and counted as enrolled. We created an enrollment flag in the EHR to identify participants who were enrolled in the cohort study.

### Data Collection

Participants completed a baseline survey about sociodemographic background, weight management practices, weight-related interactions with the health system, diet, physical activity, and quality of life using standard survey measures [10-12].

We extracted EHR data (eg, laboratory values, blood pressure, anthropometric measurements, diagnosis, and procedure codes) from each site’s PCORNet Common Data Model for all enrolled participants [7,13]. The EHR and survey data were linked for analysis.

### Statistical Methods

For this paper, we were primarily interested in describing the patterns of enrollment by recruitment modality, study site, and participant characteristics.

Anthropomorphic data were cleaned to remove unlikely values (eg, BMI ≤15 kg/m^2^ or ≥90 kg/m^2^; height ≤4 ft or ≥7 ft; weight ≤50 lbs or ≥700 lbs) prior to analyses. We identified the BMI in the EHR data closest to the enrollment date, within a maximum of 3 years, and used a 5-year window to determine comorbidity diagnoses based on International Statistical Classification of Diseases and Related Health Problems codes [14].

We used descriptive statistics (means and standard deviations for continuous measures, frequencies and proportions for nominal measures) to examine differences between the large pool of participants deemed eligible using the computable phenotype and those who enrolled in the study. We defined the “efficiency” of each recruitment strategy as the proportion of the total numbers of participants who enrolled divided by the total number of participants who were approached by the strategy (eg, total enrolled via email divided by total who were sent the email). We performed ANOVA *F* tests and chi-square tests (or Fisher exact tests, where appropriate) to determine if participant characteristics varied by recruitment modality. Because the 5 health systems preferentially designed and utilized different recruitment modalities, we were not able to assess predictors of enrollment by modality using regression models because of complete collinearity by study site. Two-sided *P*≤.05 was considered statistically significant. Analyses were performed using SAS statistical software (version 9.4; SAS Institute).

## Results

**Figure 1:**
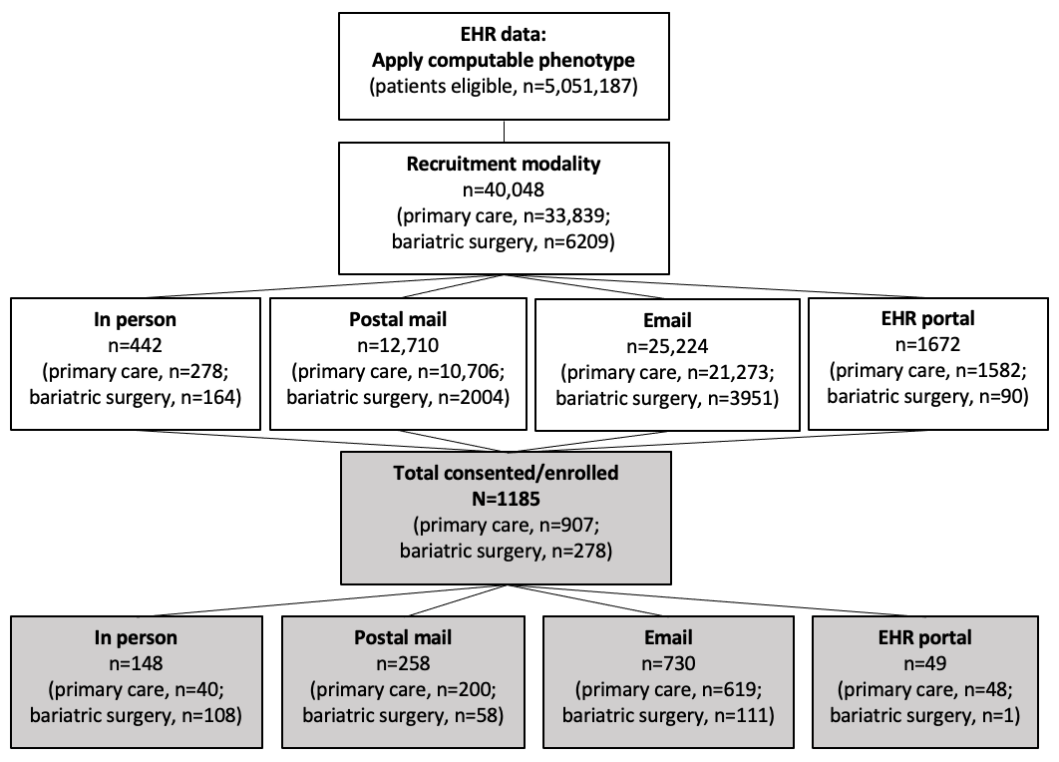
Recruitment flow of participants enrolled into the cohort from 5 health systems using 4 recruitment strategies. EHR: electronic health record.

Figure 1 shows the enrollment steps and efficiency of enrollment into the cohort by recruitment modality. We identified 5,051,187 eligible patients using the computable phenotype applied to the EHRs from 5 health systems. Based on each health systems’ own method of partnering with clinics and recruiting participants, a total of 40,048 patients (33,839 patients from primary care clinics and 6209 patients from bariatric surgery clinics) were then sent recruitment messages or approached in person. Across all sites, the patient recruitment strategies were deployed as follows: 442 (1.1%) patients in person; 12,710 (31.7%) patients by postal mail; 25,224 (63.0%) patients by email; and 1672 (4.2%) patients by EHR patient portals. A total of 1185 participants were enrolled in the cohort, with 907 (76.5%) from primary care practices and 278 (23.5%) from bariatric surgery practices. The efficiency of enrollment by recruitment strategy was by far the highest for in-person recruitment (148/442, 33.5%), followed by email (730/25,224, 2.9%) and EHR patient portal (49/1672, 2.9%), with postal mail being least efficient (258/12,710, 2.0%). Overall, 65.7% (779/1185) were enrolled through electronic messaging (email or EHR portal).

Table 1 compares the demographics and medical conditions between the 1185 participants enrolled in the cohort from primary care and bariatric surgery clinics and the 5,051,187 total patients deemed potentially eligible based on the application of the computable phenotype to their EHRs. Compared with all eligible participants, enrollees were older (aged 58.1 years in primary care clinics and 56.5 years in bariatric surgery clinics versus 43.6 years among all eligible). The bariatric surgery group had the highest proportion of women (207/278, 74.5%) compared with primary care (595/907, 65.6%) and all eligible patients (2,778,178/5,051,187, 55.0%). The proportion of Black individuals among the enrolled patient groups was smaller than that among all patients deemed eligible (81/907, 8.9% enrolled in primary care and 27/278, 9.7% in bariatric surgery versus 558,125/5,051,187, 11.1% from all patients deemed eligible). Overall, very few Hispanic participants were enrolled (14/907, 1.5% in primary care and 1/278, 0.4% in bariatric surgery) compared with the number of Hispanic individuals among the health systems’ eligible participants (127,045/5,051,187, 2.7%). Information regarding level of education was not available for nonenrolled patients; in those enrolled from either primary care or bariatric surgery clinics, more than 85% of participants had at least some college education. The mean BMI was highest for the patients from bariatric surgery clinics (34.3 kg/m^2^), followed by the patients from primary care clinics (30.2 kg/m^2^) and the total population of eligible patients (28.3 kg/m^2^). A high proportion of enrollees had hypertension (507/1185, 42.8%) and diabetes (232/1185, 19.6%), but these conditions were even more common among the large sample of eligible patients who were not enrolled (689,925/5,051,187, 52.1% with hypertension and 407,036/5,051,187, 30.7% with diabetes).

**Table 1:** Description of participants enrolled in the study cohort from primary care and bariatric surgery clinics versus all eligible participants.a

			Participants enrolled in the study cohort (N=1185)					
Participant characteristics			Primary care clinics (n=907)		Bariatric surgery clinics (n=278)		All eligible participants (n=5,051,187)	
Age (years), mean (SD)			58.1 (16.0)		56.5 (15.7)		43.6 (23.1)	
Sex (female), n (%)			595 (65.6)		207 (74.5)		2,778,178 (55.0)	
**Race^b^, n (%)**								
	American Indian/Alaska Native	3 (0.3)		1 (0.4)		10,075 (0.2)		
	Asian	1 (0.1)		4 (1.4)		79,876 (1.6)		
	Black/African American	81 (8.9)		27 (9.7)		558,125 (11.1)		
	White	788 (86.9)		234 (84.2)		3,812,584 (75.5)		
	Unknown	13 (1.4)		10 (3.6)		462,955 (9.2)		
**Ethnicity^b^, n (%)**								
	Hispanic	14 (1.5)		1 (0.4)		127,045 (2.7)		
	Not Hispanic	865 (95.4)		267 (96.0)		4,169,412 (89.8)		
	Unknown	23 (2.2)		6 (2.2)		345,957 (6.8)		
**Education level, n (%)**								
	Less than high school	6 (0.6)		3 (1.1)		N/A^c^		
	High school graduate or GED^d^	93 (10.3)		30 (10.8)		N/A		
	Some college or 2-year degree	225 (24.8)		69 (24.8)		N/A		
	College graduate	214 (23.6)		64 (23.0)		N/A		
	More than college degree	361 (39.8)		104 (37.4)		N/A		
	No response	8 (0.9)		8 (2.9)		N/A		
BMI, mean (SD)			30.2 (8.95)		34.3 (9.02)		28.3 (7.50)	
**BMI category, n (%)**								
	<18.5	7 (0.8)		0 (0.0)		227,768 (6.6)		
	18.5-25	256 (28.5)		39 (14.0)		1,047,409 (30.3)		
	25-30	260 (29.0)		62 (22.3)		998,279 (28.9)		
	30-35	158 (17.6)		60 (21.6)		630,232 (18.2)		
	35-40	96 (10.7)		41 (14.7)		310,980 (9.0)		
	>40	120 (13.4)		76 (27.3)		241,391 (7.0)		
**Comorbid health conditions, n (%)**								
	Heart failure	32 (3.5)		10 (3.6)		164,420 (12.4)		
	Hypertension	370 (40.8)		137 (49.3)		689,925 (52.1)		
	Diabetes	168 (18.5)		64 (23.0)		407,036 (30.7)		
Health care visit in last 6 months, n (%)			707 (77.9)		213 (76.6)		887,235 (67.0)	

^a^No statistical testing was performed because of overlap between the eligible group and those enrolled in the cohort.

^b^For participants enrolled in the cohort, race and ethnicity information was self-reported; for nonenrolled participants, these data were obtained from the electronic health record.

^c^N/A: not available.

^d^GED: general education degree.

**Figure 2:**
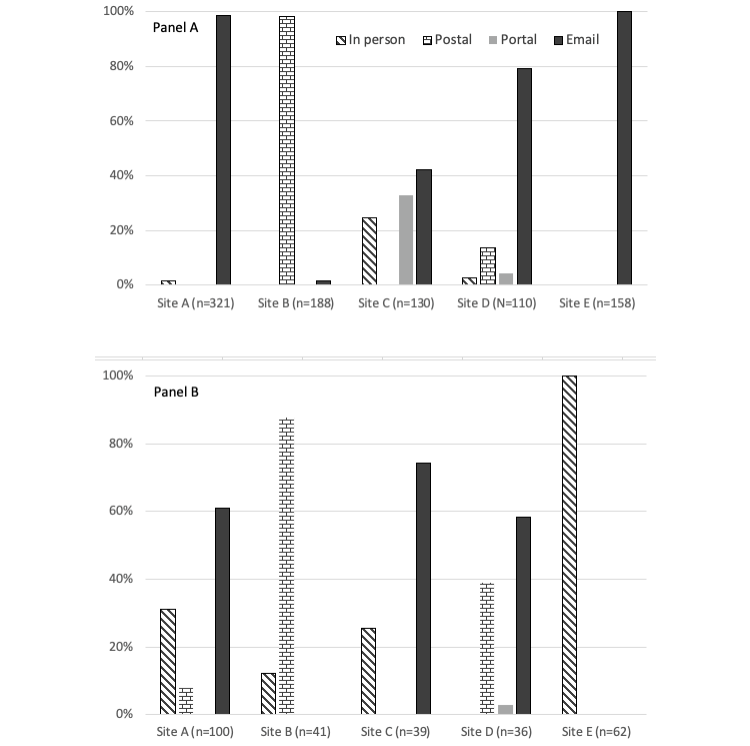
Distribution of participants by recruitment site (sites A to E) and recruitment strategy. (A) Primary care clinic participants (n=907). (B) Bariatric surgery clinic participants (n=278).

Figure 2 demonstrates the distribution of participants enrolled by strategy and recruitment site from the 5 health systems. Because of differences in research policies, staffing, and capabilities, each health system differed in the number of recruitment strategies used in the primary care and bariatric surgery settings. Table 2 shows the characteristics of enrolled cohort participants by recruitment strategy. The proportion of Black patients who were recruited using EHR patient portals (8/49, 16.3%) and email (80/730, 11.0%) was higher than that with in-person (7/148, 4.7%) and postal (13/258, 5.0%) strategies. We found no statistically significant differences in participants’ level of education or comorbid health conditions between the 4 different recruitment strategies.

**Table 2:** Description of enrolled participants (N=1185) by recruitment strategy.

		Recruitment strategy					
Participant characteristics		In-person (n=148)	Postal (n=258)	Portal (n=49)	Email (n=730)	*P* value	
Age (years), mean (SD)		58.6 (16.0)	59.0 (14.6)	60.0 (12.9)	57.1 (15.9)	.22^a^	
Sex (female), n (%)		114 (77.0)	165 (64.0)	33 (67.4)	540 (74.0)	.007^b^	
**Race, n (%)**						<.001^c^	
	American Indian/Alaska Native	1 (0.7)	1 (0.4)	0 (0)	2 (0.3)		
	Asian	2 (1.4)	0 (0)	3 (6.1)	7 (1.0)		
	Black	7 (4.7)	13 (5.0)	8 (16.3)	80 (11.0)		
	White	136 (91.9)	233 (90.3)	35 (71.4)	618 (84.7)		
	Other	0 (0.0)	6 (2.3)	0 (0)	14 (1.9)		
**Ethnicity, n (%)**						.69^c^	
	Hispanic	1 (0.7)	5 (1.9)	0 (0)	9 (1.2)		
	Not Hispanic	142 (95.9)	249 (96.5)	49 (100)	692 (94.8)		
**Education level, n (%)**						.03^c^	
	Less than high school	1 (0.7)	5 (6.1)	1 (2.0)	0 (0)		
	High school graduate or GED^d^	12 (8.1)	25 (9.7)	2 (4.1)	78 (10.7)		
	Some college or 2-year degree	37 (25.0)	67 (26.0)	11 (22.4)	181 (24.8)		
	College graduate	39 (26.4)	61 (23.6)	7 (14.3)	172 (23.6)		
	More than college degree	58 (39.2)	101 (39.1)	28 (57.1)	290 (39.7)		
BMI, mean (SD)		32.3 (8.2)	30.7 (7.9)	31.2 (9.0)	31.4 (9.0)	.20^a^	
**Comorbid health conditions, n (%)**							
	Heart failure	7 (4.7)	6 (2.3)	1 (2.0)	28 (3.8)	.58^c^	
	Hypertension	62 (41.9)	110 (42.6)	21 (42.9)	315 (43.2)	.99^b^	
	Diabetes	32 (21.6)	50 (19.4)	12 (24.5)	138 (18.9)	.50^b^	
Visit to health care provider in last 6 months, n (%)		101 (68.2)	190 (73.6)	38 (77.6)	581 (79.6)	.01^b^	
Primary care clinic participant, n (%)		40 (27.0)	200 (77.5)	48 (98.0)	619 (84.8)	<.001^b^	
Bariatric surgery clinic participant, n (%)		108 (73.0)	58 (22.5)	1 (2.0)	111 (15.2)		
**Site, n (%)**						<.001^c^	
	A	36 (24.3)	8 (3.1)	0 (0)	377 (51.9)		
	B	5 (3.4)	221 (85.7)	0 (0)	3 (0.4)		
	C	42 (28.4)	0 (0)	43 (87.8)	84 (11.6)		
	D	3 (2.0)	29 (11.2)	6 (12.2)	108 (14.9)		
	E	62 (41.9)	0 (0)	0 (0)	158 (21.7)		

^a^ANOVA *F* test.

^b^Chi-square test.

^c^Fisher exact test.

^d^GED: general education degree.

## Discussion

This study reports the experience of the PaTH CRN’s recruitment of patients from primary care and bariatric surgery clinics from 5 health systems into a study cohort. Even though the in-person recruitment had the greatest efficiency (33.5%), this strategy required research staff to identify when patients had upcoming appointments and to be available on site. Ultimately, only 442 patients were approached in person and 148 enrolled. The majority of participants were recruited using email (730/1185, 61.6%). Although email recruitment required less staff research time, this strategy was limited by having patients’ email addresses recorded and available in the EHRs. The secure patient portal was a strategy employed by 2 health systems and had a recruitment efficiency comparable to email (2.9% of those who received a message enrolled in the study). The electronic recruitment strategies may be particularly desirable during times when research staff cannot be present in the clinic, such as under COVID-19–related restrictions.

Traditionally, researchers have relied on postal mailings, newspaper or radio advertising, or random-digit dialing methods for survey-based research as well as for recruitment into clinical trials [15]. EHRs provide an avenue for screening and then directly targeting recruitment at potentially eligible patients for research studies [4,15]. Recruitment of patients from EHRs has the potential to be more inclusive by including patients who might be sicker and those who are undergoing care in real-world health care systems [5,16]. However, no method of recruitment is without the potential for selection biases. With EHR-based recruitment, there are concerns about external validity or generalizability to nonpatient or health care populations, particularly for prevention-oriented studies [17]. In addition, patients with reduced access to care or those who are not regularly followed in the health care setting may be less likely to be approached in person (ie, at the time of a clinic visit) or to receive recruitment messages when they do not have an email address or access the patient portal. While 90% of US adults now use the internet, older adults and Black citizens still show lower rates of internet access [18], but technology usage rates are increasing [19]. A recent study by Walker and colleagues [18] showed that fewer Black patients and patients over the age of 70 had access to an inpatient portal. Notably, in our study, which used a combination of recruitment strategies, approximately 9% of enrolled participants identified as Black compared with 11% among patients identified as potentially eligible. In fact, researchers could leverage the EHR to specifically target patient populations by demographics or medical diagnosis (eg, by race, ethnicity, age, or rare health conditions [20,21]).

Despite the increasing need for effective and less staff-intensive methods of recruitment for clinical trials and surveys, few studies have compared recruitment strategies [22,23]. One study reported and compared response rates from various recruitment methods (postal survey, postal invitation to complete an internet survey, and postal invitation for a telephone survey) for an environmental survey but did not include EHR-based recruitment methods [23]. The study showed the highest response rate (30%) for the telephone survey [23]. The EHR provides a new way to identify potential participants for research studies by applying electronic eligibility criteria to sometimes very large pools of potentially eligible patients [24].

A systematic review by Lai and Afseth [25] assessed the effectiveness and efficiency of EHR-based recruitment methods. They identified 13 articles, of which 11 reported recruitment efficiency and most used alerts sent directly to physicians or staff to notify of participant eligibility [25]. EHRs have multiple functionalities to support recruitment [26]. In our study, we utilized the EHR for several purposes: to generate lists of potentially eligible patients using a computable phenotype, to obtain postal mailing addresses and email addresses, and to send recruitment invitations using the secure patient portal at 2 sites. Some academic research centers have designed patient portal recruitment services to enable, but also limit, recruitment using the portal for certain approved studies [1,21,27,28]. In a 2019 single-institution study that included 13 separate EHR-based recruitment strategies using the patient portal recruitment service, the average response rate for patient portal messages was 2.9%, which was the same as our enrollment rate for both email and patient portal recruitment [21]. Interestingly, we offered no compensation to patients to enroll in our study, yet the studies reviewed by Miller et al [21] did offer compensation.

Although the computable phenotype enabled our teams to identify a very large number of patients who were potentially eligible, each health system designed its own outreach methods to patients by targeting specific clinical sites, thereby greatly reducing the potential number of patients that could have been contacted about this research opportunity. Importantly, institutional review boards at each site prohibited the “cold calling” of patients (ie, directly contacting potential research participants based on prior knowledge of the patients’ health information in the absence of a treatment or clinical relationship [29]). Therefore, each site partnered with primary care and bariatric surgery providers, who approached the patients about the study first, either with their signature on the recruitment invitation or through an in-person introduction. However, a recent landscape analysis by McHugh and colleagues [29] highlighted that this universally applied “ban on cold calling” could impose a gatekeeping function and potentially reduce patient autonomy, decreasing access to research and risking the introduction of selection bias into research studies. They suggested alternative approaches to ensuring patient privacy [29], with the goal of broadening access to health research participation [15].

We identified several limitations of this study. First, this was a 5-center, multisite study and the deployed recruitment strategies were dependent on the norms for research recruitment at each site, with the implication that not all samples were directly comparable by site or by strategy. For example, 3 sites did not allow research recruitment using the EHR patient portal and 1 site did not have access to email addresses in the EHRs. Because of the differences in clinical populations’ demographics between sites, there was high correlation between the site’s choice of recruitment methods and the patients who were recruited, limiting our ability to draw conclusions about whether specific recruitment modalities are more (or less) effective for specific racial/ethnic groups or different age categories. Second, the overall response rate was low; however, it is very comparable to response rates from other studies that relied on EHR-based or email-based recruitment. We were able to assess for selection biases by comparing those patients who were deemed eligible with those who enrolled in the study. We showed lower uptake among Latinx patients, but this was in part because the consent and survey were limited to participants able to read in English. Third, although this study was low burden for participants, as they consented to having the research team review medical records and complete a 20-minute online survey, we did not offer any compensation for their time, which could have limited enrollment. Therefore, we are not able to draw conclusions about how effective our recruitment strategies would be for more intensive studies, for studies not using online data collection, or for studies offering participant incentives. Fourth, we were unable to estimate cost or cost-effectiveness of the recruitment strategies or describe in detail the staffing time or corresponding resources involved for each health system, such as the effort for the health informatics team to support EHR-based research or the time for research staff to conduct in-person clinic-based recruitment. Fifth, recruitment for this study occurred approximately 4 years ago (2015-2016) and it is possible that response rates and methods for electronic recruitment have improved over time. Sixth, the 5 health systems were all based in the mid-Atlantic region of the United States, which could limit the generalizability of these findings to other regions of the country and outside the United States.

The major implication of our study is to inform the selection of recruitment strategies for the design of future cohort studies, utilizing the capabilities of the modern-day EHR system. We anticipate that other researchers could find this information useful in the design of their recruitment strategies and to estimate the expected yields from “low touch” strategies that require less personnel contact with potential participants. As health systems and institutional review boards become more comfortable and familiar with EHR-based recruitment, it will be possible to achieve greater consistency between the recruitment processes and even sample selection to reduce biases across systems. Ultimately, to facilitate EHR-based research across multiple settings (eg, inpatient and ambulatory), large health systems will need to invest in infrastructure to support and link smaller clinical centers and subsidize their use of a common EHR. An example of a successful academic and community-based network is the OCHIN network, which provides a research infrastructure using EHR data from a national network of smaller community health centers [30].

In conclusion, we recruited a total of 1085 patients from primary care and bariatric surgery clinics to complete a survey and participate in an EHR-based cohort study using 4 recruitment methods. The greatest recruitment yield was achieved using the email-based method, but the greatest efficiency resulted from in-person recruitment. Implementation of low-resource recruitment approaches has important implications for future patient-centered studies in health system settings.

## Abbreviations

CRN: Clinical Research Network
EHR: electronic health record
PCORI: Patient-Centered Outcomes Research Institute
PCORnet: National Patient-Centered Research Network
